# A silver-nanoparticle/cellulose-nanofiber composite as a highly effective substrate for surface-enhanced Raman spectroscopy

**DOI:** 10.3762/bjnano.10.126

**Published:** 2019-06-24

**Authors:** Yongxin Lu, Yan Luo, Zehao Lin, Jianguo Huang

**Affiliations:** 1 Department of Chemistry, Zhejiang University, Hangzhou, Zhejiang 310027, P. R. China; 2 Shaoxing Test Institute of Quality and Technical Supervision, Shaoxing, Zhejiang 312071, P. R. China

**Keywords:** cellulose nanofiber, composites, nanoarchitectonics, silver nanoparticle, surface-enhanced Raman spectroscopy

## Abstract

A highly active surface-enhanced Raman scattering (SERS) substrate was developed by facile deposition of silver nanoparticles onto cellulose fibers of ordinary laboratory filter paper. This was achieved by means of the silver mirror reaction in a manner to control both the size of the silver nanoparticles and the silver density of the substrate. This paper-based substrate is composed of a particle-on-fiber structure with the unique three-dimensional network morphology of the cellulose matrix. For such a SERS substrate with optimized size of the silver nanoparticles (ca. 70 nm) and loading density of silver (17.28 wt %), a remarkable detection limit down to the sub-attomolar (1 × 10^−16^ M) level and an enhancement factor of 3 × 10^6^ were achieved by using Rhodamine 6G as the analyte. Moreover, this substrate was applied to monitor the molecular recognition through multiple hydrogen bonds in between nucleosides of adenosine and thymidine. This low-cost, highly sensitive, and biocompatible paper-based SERS substrate holds considerable potentials for the detection and analyses of chemical and biomolecular species.

## Introduction

In the last decades, impressive advances have been made in nanoscience and nanotechnology both in fundamental research and practical applications. The basis for nanoscience and nanotechnology, the construction of functional materials with specific structural features at the nanoscale, is now stepping up from “nanofabrication” to “nanoarchitectonics” [1]. Nanoarchitectonics as a novel paradigm to create specific materials by assembling the corresponding nanoscale building blocks was first proposed by M. Aono and co-workers in the year 2000 [2–3]. The concept has been recently extended extensively and deepened systematically by K. Ariga and his colleagues [4–14]. Various functional units ranging from atoms and molecules to polymers, biomacromolecules and nanoscale objects are employed for the construction of specific nanoarchitectures by various chemical methodologies such as self-organization and layer-by-layer self-assembly [7,15–17]. In particular, layer-by-layer self-assembly holds significant potentials for the fabrication of a large variety of functional nanoarchitectures [18–25]. It has been demonstrated that the different nanoarchitectures developed so far have prominent application potentials in the areas of sensors and devices [26–30], catalysts [17,31–32], energy materials [16,32–33], as well as bio-oriented applications [34–42]. In the current work, a functional nanoarchitecture composed of silver nanoparticles anchored on cellulose nanofibers was fabricated, which is shown to be a highly effective substrate for surface-enhanced Raman spectroscopy (SERS).

SERS, a powerful molecular spectroscopy method, is widely used in the trace detection and characterization of various chemical and biological substances where the substrates are crucial for obtaining an enhanced Raman signal [43–45].

The Raman signal of SERS is enhanced remarkably in the “hot spots” that are generated in the nanogaps of plasmonic metal nanoparticles (e.g., Au, Ag and Cu) through the amplification of the electromagnetic field caused by localized surface plasmon resonance [46]. In order to create more nanogaps and to generate more hot spots to improve the SERS effect, a number of nanostructures based on metal particles were prepared by different methods, such as thermal evaporation [47], electrospray [48], inject printing [49], successive ionic layer absorption and reaction (SILAR) [50], and photochemical methods [51]. However, there are still challenges regarding the facile fabrication of the SERS substrates with high spectroscopic performance.

Regarding SERS substrates, the choice of the substances employed on which the metal nanoparticles are deposited influences both the collection efficiencies and detection sensitivities. Cellulose, such as laboratory filter paper and bacterial nanocellulose, have been considered as superior candidates for the fabrication of SERS substrates with silver nanoparticles, due to their low cost, wide availability, as well as flexibility, portability and biodegradability [52–54]. The high surface density of hydroxy groups in cellulose results in a sufficient stability of the deposited silver nanoparticles via Ag–O bonding [52]. Moreover, the unique three-dimensionally cross-linked porous structure and the hierarchical morphologies at micro- and nanoscale of bulk cellulose lead to the creation of more hot spots by the loaded silver nanoparticles, and therefore, to higher SERS enhancement. Furthermore, cellulose materials such as filter paper are structurally porous, physically flexible and hydrophilic, which allows for a facile and efficient collection of the analytes from solution media. Hence, a number of works have been reported concerning the fabrication of SERS substrates by deposition of silver particles onto cellulose filter paper by means of the silver mirror reaction [55–59]. In order to ensure the effective loading of silver particles on the cellulose fiber surfaces, relatively high reaction temperatures (above 45 °C) [55–58] or strong reducing agents (such as formaldehyde) [59] were applied in the reactions, which resulted in rather large sizes and excessive loading densities of the silver particles. Eventually this led to somewhat low sensitivities of the substrates, because detection limits at the sub-attomolar level could not be achieved.

In the present work, a SERS substrate was fabricated by the deposition of silver nanoparticles (Ag-NPs) onto the surfaces of the cellulose nanofibers (NFs) in ordinary laboratory filter paper by means of the one-step silver mirror reaction. Both size and density of the of the silver nanoparticles on the substrates could be controlled. This paper-based silver-nanoparticle/cellulose-nanofiber (Ag-NP/cellulose-NF) showed a very good SERS performance. In the optimized case, the detection limit of Rhodamine 6G (R6G) was as low as 1 × 10^−16^ M (sub-attomolar level) with just a small droplet of solution needed (10 µL). This is superior to some of the reported works mentioned above [55–59]. The SERS substrate was also applied to monitor the molecular recognition through multiple hydrogen bonds between adenosine and thymidine. This paper-based SERS substrate could hold potential in the detection of trace amounts of analytes and for the spectroscopic study of biomolecules.

## Results and Discussion

### Characterization of the Ag-NP/cellulose-NF composite

The silver-nanoparticle/cellulose-nanofiber SERS substrates were fabricated by deposition of silver nanoparticles onto the surfaces of the cellulose nanofibers of ordinary laboratory filter paper by the silver mirror reaction. As described in the Experimental section, the sizes of the silver nanoparticles and the final silver densities of the substrates were adjusted by varying the deposition time. The substrates obtained after deposition times of 2, 4, 6, 8, and 10 min are denoted as sample Ag-NP/cellulose-NF–A, B, C, D, and E, respectively.

**Figure 1:**
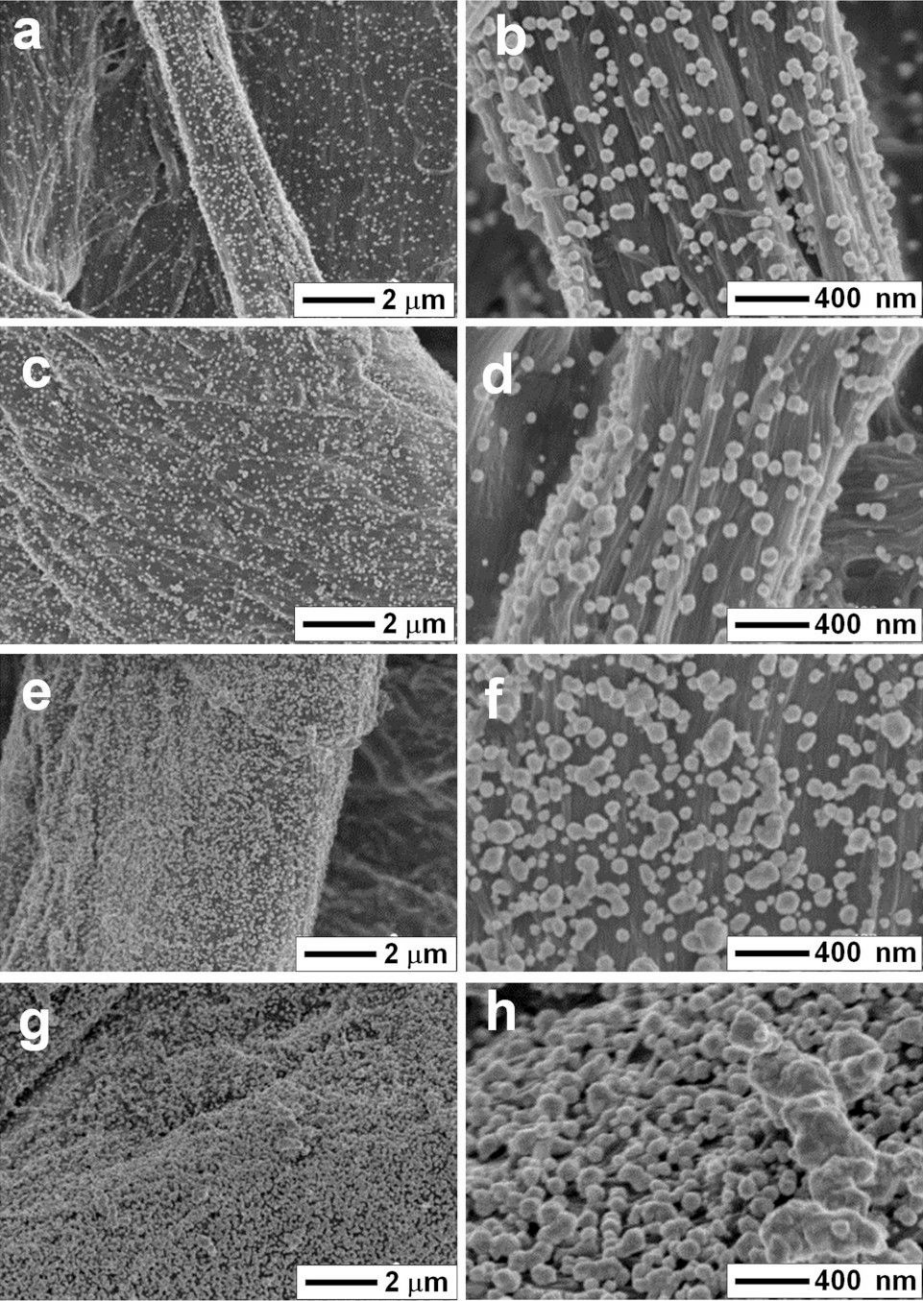
FE-SEM micrographs of the paper-based SERS substrates Ag-NP/cellulose-NF–A (a,b), B (c,d), C (e,f), and D (g,h), which were fabricated with reaction times of 2, 4, 6, and 8 min, respectively.

The field-emission scanning electron microscopy (FE-SEM) images of the Ag-NP/cellulose-NF composite sheets in Figure 1 show that the surfaces of the cellulose fibers are decorated with silver particles. The comparison with SEM images of bare filter paper (Supporting Information File 1, Figure S1) shows that the structural integrity of the filter paper was not affected by the deposition process. With increasing reaction time, the particle size increased gradually; and excessive reaction time resulted in much larger particle sizes and obvious aggregation of the silver particles, as seen for the samples Ag-NP/cellulose-NF–D (Figure 1g,h) and –E (Supporting Information File 1, Figure S2), where the cellulose fibers are fully coated with silver layers. In the samples Ag-NP/cellulose-NF–A, –B and –C (Figure 1a–f), silver nanoparticles with an average size of 46.5, 70.2, and 75.8 nm (Supporting Information File 1, Figure S3a,c,e), respectively, are uniformly anchored on the cellulose fibers. According to energy-dispersive X-ray (EDX) analyses, the silver contents of the corresponding samples were 0.49, 9.61, and 17.28 wt %, respectively (Supporting Information File 1, Figure S3b,d,f). As demonstrated in Figure 1f, more nanogaps between the silver nanoparticles exist in the substrate Ag-NP/cellulose-NF–C, which would be beneficial for the enhanced SERS effect.

**Figure 2:**
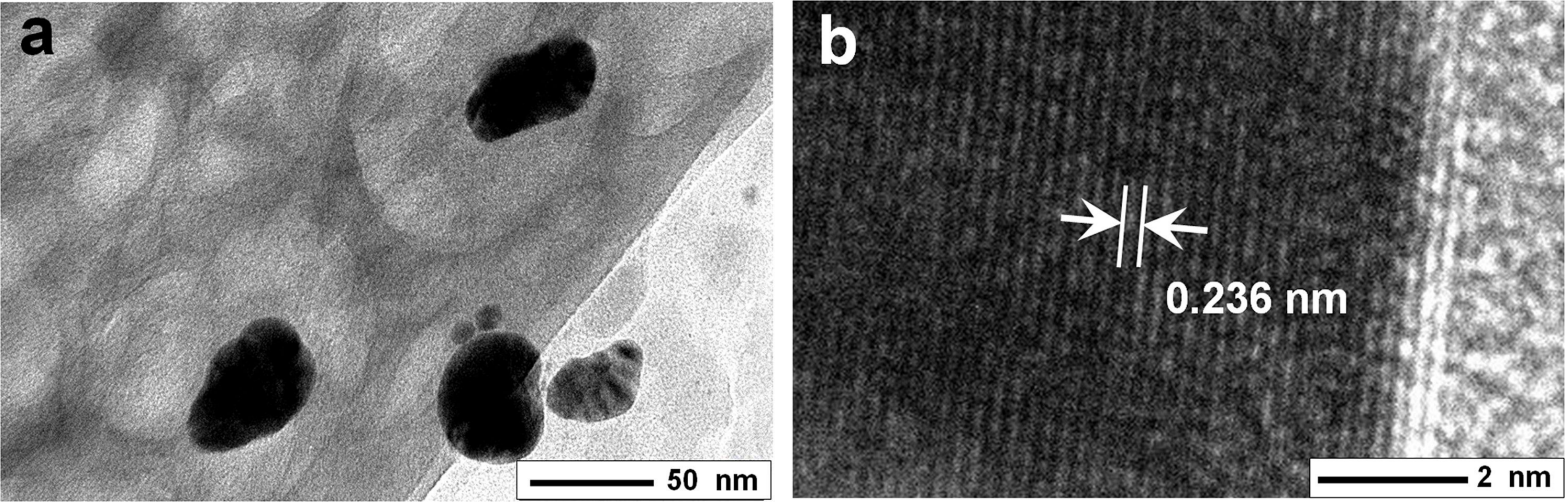
TEM image of Ag-NP/cellulose-NF–C (a), and HR-TEM image of an individual silver nanoparticle showing the lattice of metallic silver (b).

Figure 2a shows the transmission electron microscopy (TEM) image of the sample Ag-NP/cellulose-NF–C showing the silver nanoparticles anchored on the cellulose fibers. The amount of the silver nanoparticles observed is much less than that of the FE-SEM image (Figure 1f), which is because some nanoparticles were apparently lost from the as-prepared sample during the preparation procedure of the specimen, as noted in the Experimental section. The high-resolution TEM (HR-TEM) image of an individual silver nanoparticle is displayed in Figure 2b, the lattice spacing of 0.236 nm observed is corresponding to the (111) plane of metallic silver [60], confirming the formation of the silver nanoparticles.

**Figure 3:**
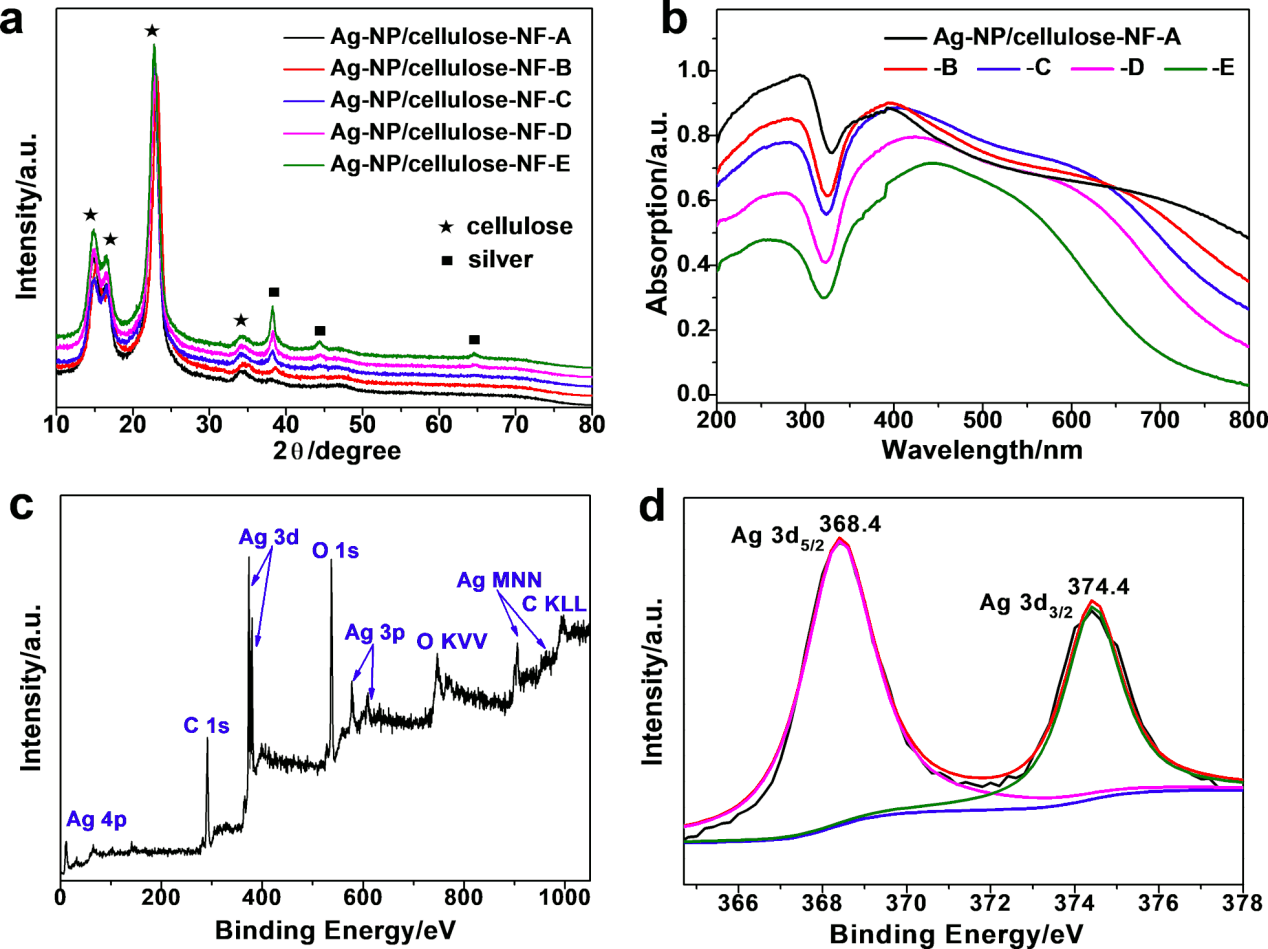
X-ray diffraction patterns (a) and diffuse reflectance UV–vis spectra (b) of Ag-NP/cellulose-NF–A, B, C, D, and E. X-ray photoelectron spectroscopy (XPS) survey spectrum (c) and high-resolution XPS spectrum of the Ag 3d region (d) of sample Ag-NP/cellulose-NF–C.

Figure 3a shows the X-ray diffraction (XRD) patterns of the prepared Ag-NP/cellulose-NF composites. Two series of diffraction peaks were observed. The ones located at 2θ = 15.0°, 16.5°, 22.8°, and 34.1° are ascribed to the (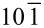), (101), (002), and (040) planes of crystalline cellulose, respectively [61]; and the other ones located at 2θ = 38.1°, 44.3°, and 64.4° are assigned to the (111), (200), and (220) planes of metallic silver phase, respectively [60]. It is noticed that the diffraction peak intensities of metallic silver increased along with the increment of the silver content in the samples, which agrees well with the FE-SEM results. The reflectance UV–vis spectra of the samples are presented in Figure 3b. No obvious absorption band was observed for the bare cellulose filter paper (Supporting Information File 1, Figure S4). For sample Ag-NP/cellulose-NF–A, the strong surface plasmon resonance absorption band of silver nanoparticles was observed at around 400 nm. With increasing size of the silver nanoparticles, this band gradually broadened and red-shifted to 450 nm for sample Ag-NP/cellulose-NF–E. It is known that, along with the increment of the silver nanoparticle sizes, the corresponding surface plasmon resonance band red-shifts to higher wavelengths [62]. It is also seen that every spectrum shows a shoulder band between 350 and 400 nm, which becomes more prominent with increasing silver nanoparticle size. This is because multipole transitions of surface plasmons become more prominent with the increment of the particle size [62]. These results are in accordance with the electron microscopy observations discussed above.

The X-ray photoelectron spectroscopy (XPS) survey spectrum of sample Ag-NP/cellulose-NF–C is shown in Figure 3c, showing the distinct peaks of carbon, oxygen and silver. Figure 3d shows the corresponding high-resolution spectrum of the Ag 3d region, where the two peaks located at 368.4 and 374.4 eV are attributed to the binding energies of Ag 3d_5/2_ and Ag 3d_3/2_ of metallic silver, respectively [60]. This result indicates metallic silver in the as-prepared paper-based SERS substrate, which is in good agreement with the afore-mentioned characterizations.

### SERS performance of the Ag-NP/cellulose-NF substrate

**Figure 4:**
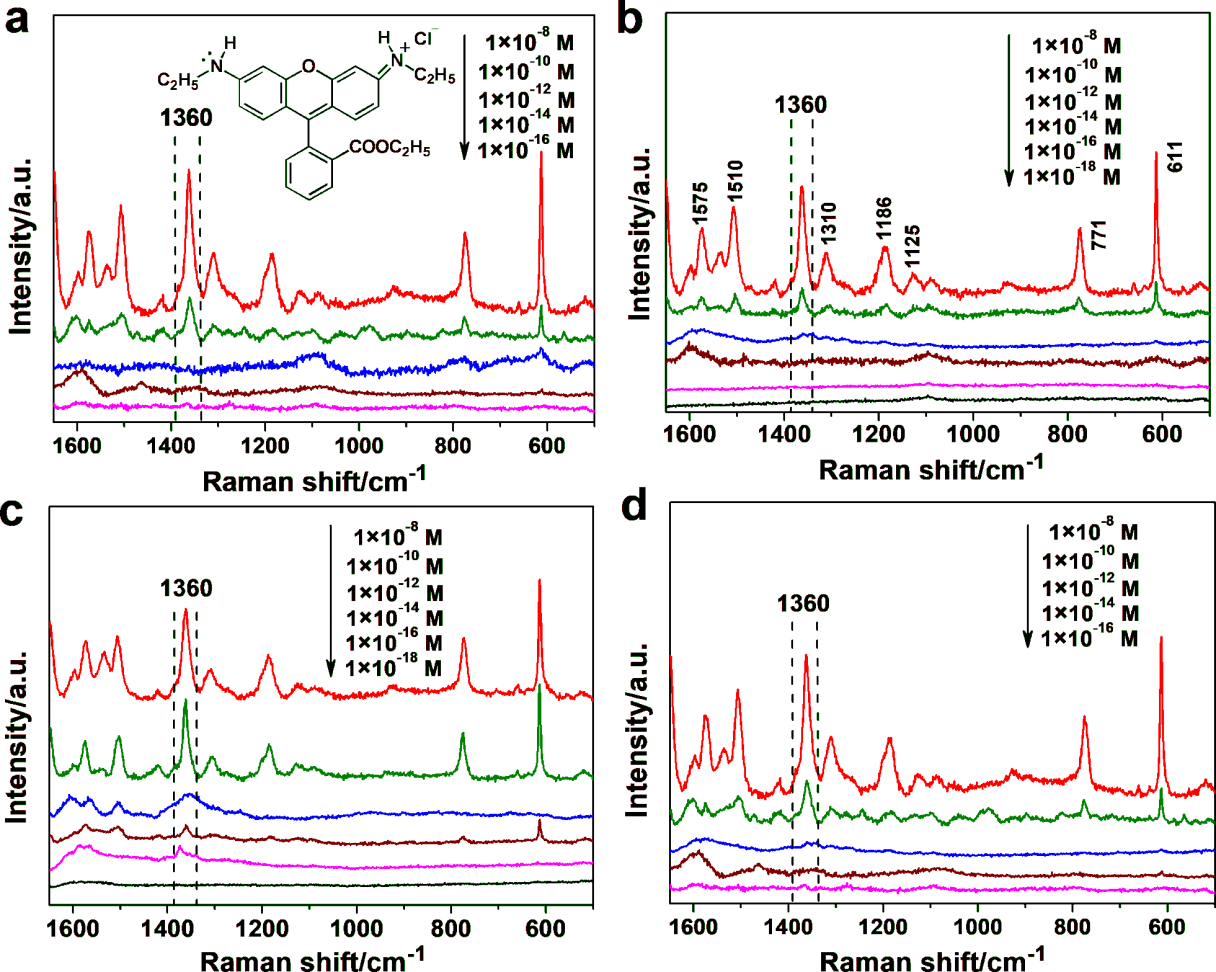
SERS spectra of Rhodamine 6G (R6G) at different concentrations obtained by employing Ag-NP/cellulose-NF–A (a), –B (b), –C (c), and –D (d) composite sheets as the substrates. The characteristic band at 1360 cm^−1^ was used as the reference, and the SERS measurements were acquired from four randomly selected locations on each of the samples. The inset of (a) is the molecular structure of R6G.

The performance of the Ag-NP/cellulose-NF composite sheets as SERS substrates was investigated by using Rhodamine 6G (R6G, inset of Figure 4a) as probe molecule. Neither the filter paper itself nor the pure Ag-NP/cellulose-NF substrate gave any spectral peak in the wavenumber region measured (Supporting Information File 1, Figure S5). R6G is employed as the model analyte due to its strong affinity to silver particles and its distinct Raman fingerprint [53]. All samples gave the characteristic Raman scattering bands of R6G at relatively high concentrations (Figure 4; Supporting Information, Figure S6). As marked in Figure 4b, the bands located at 611, 771, and 1125 cm^−1^ are assigned to the C–C–C ring in-plane, out-of-plane bending, and C–H in-plane bending vibrations, respectively; and those at 1186, 1310, 1360, 1510, and 1575 cm^−1^ are associated with the totally symmetric modes of in-plane C–C–C stretching vibrations [63–64].

It is seen from the spectra in Figure 4a that the intensities of the Raman signals decreased along with the decrement of the concentrations of R6G. The most intense Raman band at 1360 cm^−1^ was adopted to compare the detection limit of the series of substrates.

The substrate Ag-NP/cellulose-NF–C exhibited the highest SERS enhancement, a detection limit as low as 1 × 10^−16^ M was achieved (Figure 4c). It is known that the SERS signals mainly result from the plasmon coupling of the metal particles, which depends considerably on the density and morphology of the particles. For substrates Ag-NP/cellulose-NF–A and –B, the detection limits are 1 × 10^−10^ M and 1 × 10^−12^ M, respectively (Figure 4a,b), which is due to the large distance between the neighboring silver nanoparticles. And, as for the samples Ag-NP/cellulose-NF–D and –E, the corresponding detection limits are 1 × 10^−12^ M (Figure 4c) and 1 × 10^−10^ M (Supporting Information File 1, Figure S6), respectively. This is because, as seen from the FE-SEM images of the two substrates (Figure 1h; Supporting Information File 1, Figure S2), large aggregates of silver particles were formed during the prolonged reaction time, which resulted in a reduced number of hot spots. It can thus be concluded that, sample Ag-NP/cellulose-NF–C possesses the optimal loading density and morphology of the silver nanoparticles for SERS application. This SERS substrate is usable for the trace detection of the analyte.

**Figure 5:**
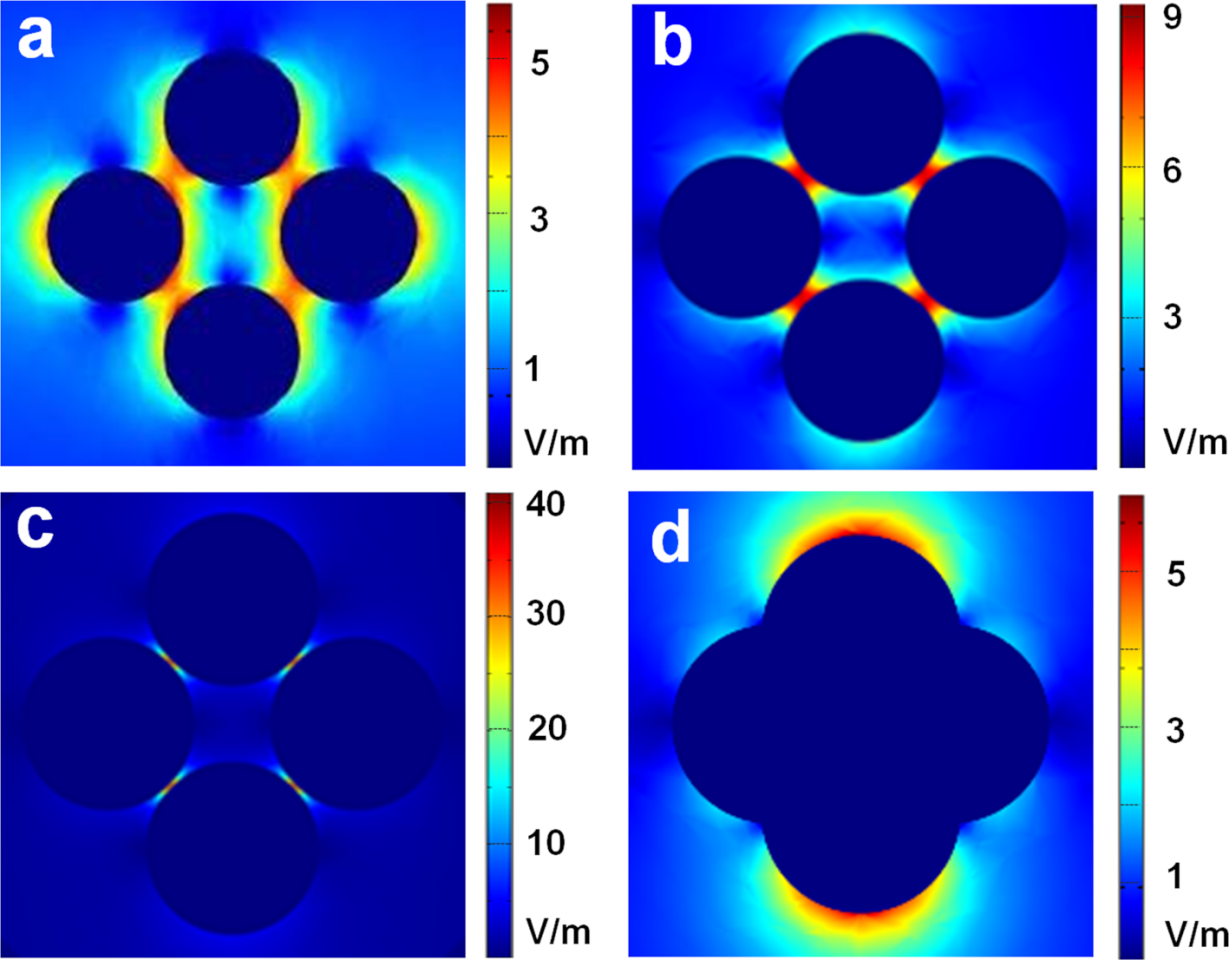
Electric field intensity distributions (indicated by the color bar) of the silver nanoparticles (diameter 70 nm) with different inter-particle spacings of 15 nm (a), 5 nm (b), 1 nm (c), and aggregated particles (d) at an excitation wavelength of 514 nm.

In order to better understand the high SERS activity of the substrate Ag-NP/cellulose-NF–C, finite element method (FEM) modeling was performed to investigate the localized electric field intensity (*E*_max_) of the silver nanoparticles (diameter 70 nm) with different inter-particle spacings. The obtained electric field intensity distributions are shown in Figure 5. The maximum values of the electric field intensities for inter-particle spacings of 15, 5, and 1 nm are 5.7, 9.2, and 40.7 V/m, respectively, and the value for the aggregated nanoparticles is 4.8 V/m. This result indicates that a smaller inter-particle distance of the silver nanoparticles leads to stronger electric fields. The sample Ag-NP/cellulose-NF–C shows a small inter-particle distance and, therefore, exhibits the best SERS activity. According to the fourth-power dependence of the enhancement factor on the electric field intensity, the enhancement factor of this substrate was estimated to be ca. 3 × 10^6^.

Compared with previously reported cellulose-based SERS substrates, our current substrate shows a better SERS activity. For example, the bacterial nanocellulose adsorbed with gold nanoparticles in the form of a hydrogel had a detection limit of 1 × 10^−9^ M for R6G [54], the Au–Ag bimetallic microfluidic SERS sensor had a detection limit of 1 × 10^−13^ M for the same analyte [64]; and a detection limit of 1 × 10^−6^ M was offered by a hybrid substrate composed of cellulose nanofibrils and silver nanoprisms [65]. Two further examples are the silver dendrite decorated filter membrane and the silver nanoparticle decorated plasmonic paper, which both had a detection limit for R6G of 1 × 10^−11^ M [56,66]. The current paper-based Ag-NP/cellulose-NF SERS substrate achieved a remarkable detection limit at the sub-attomolar (1 × 10^−16^ M) level, which offers an outstanding potential for the detection and analyses of trace amounts of analytes.

**Figure 6:**
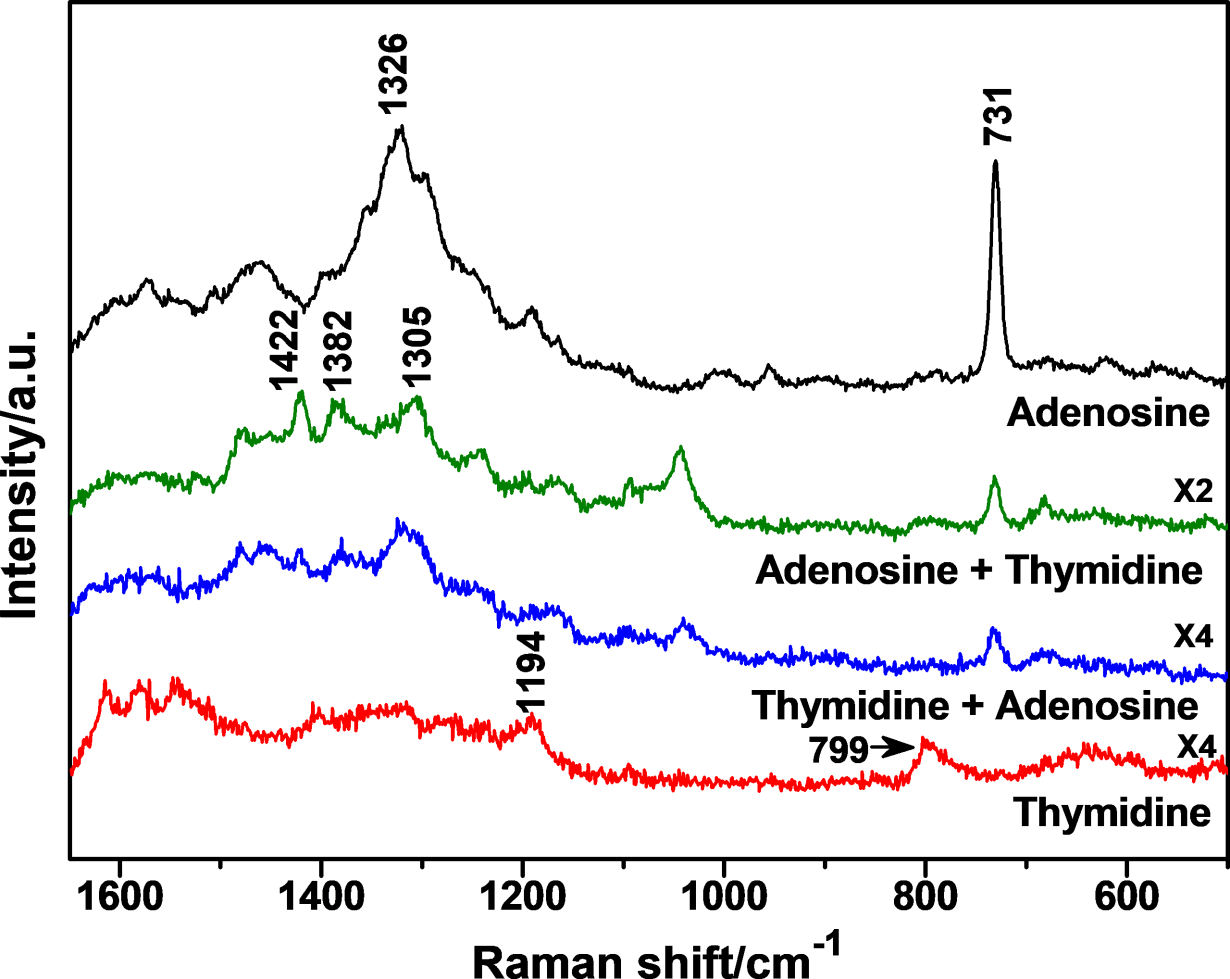
SERS spectra of adenosine and thymidine (both 10 µM), and the surface mixture of adenosine and thymidine obtained by employing the paper-based substrate Ag-NP/cellulose-NF–C.

This active paper-based SERS substrate was employed to detect the molecular recognition through multiple hydrogen bonds between nucleosides to test its potential in monitoring biomolecules. Figure 6 shows the Raman spectra obtained from adenosine and thymidine, measured before and after the molecular recognition between the two nucleosides on the substrate. Compared to the Raman spectra of the powder samples of adenosine and thymidine (Supporting Information File 1, Figure S7), different spectral features were observed. For adenosine (Figure 6, black curve), the strong scattering band located at 731 cm^−1^ is attributed to the ring breathing vibration of the adenine moiety, and the band at 1326 cm^−1^ is assigned to the stretching vibration of C–N and the bending vibration of C–H [67]. For thymidine (Figure 6, red curve), the weak bands at 799 and 1194 cm^−1^ are due to the ring breathing vibration and C–CH_3_ stretching vibration of the thymine moiety, respectively [68]. It was noticed that the signal intensities of thymidine are much weaker than those of adenosine. This is because the amino group contained in the adenine moiety of adenosine leads to a much stronger interaction between the molecule and the surface of the silver nanoparticle. After the molecular recognition process occurred for the two nucleosides, similar spectra (Figure 6, green and blue curves) were observed for both addition sequences (see Experimental section). The relative intensity of the band at 731 cm^−1^ of adenosine decreased, the band at 799 cm^−1^ of thymidine disappeared, and the initial band at 1326 cm^−1^ of adenosine became much weaker and red-shifted to 1305 cm^−1^. Moreover, two new bands at 1382 and 1422 cm^−1^ attributed to the ring breathing vibrations of the thymine moiety of thymidine raised. These spectral features, which depend on the specific molecular orientation of the analyte on the silver surface, indicate the formation of complementary hydrogen bonds between the adenine and thymine moieties of the two nucleosides. This result demonstrates the potential of the SERS substrate for biomolecular analyses.

## Conclusion

In summary, a paper-based SERS substrate with high activity was fabricated by the facile deposition of silver nanoparticles onto the cellulose fibers of laboratory filter paper. That is, the substrate exhibits a silver-nanoparticle/cellulose-nanofiber nanoarchitecture. Because of the optimized loading density and morphology of the deposited silver nanoparticles, as well as the unique structure of the cellulose matrix, this substrate exhibits a high SERS activity. A sub-attomolar-level detection limit is achieved for the detection of Rhodamine 6G. The substrate may be applicable for facile and sensitive analyses of chemical and biomolecular substances using Raman spectroscopy.

## Experimental

### Chemicals

Silver nitrate (AgNO_3_), β-ᴅ-glucose and concentrated ammonia were bought from Sinopharm Chemical Reagent Co., Ltd. (Shanghai, China); sodium hydrate (NaOH) was purchased from Shanghai Titanchem Co., Ltd. (Shanghai, China); Rhodamine 6G (R6G, 98.5%) was obtained from J&K Chemical Ltd. (Shanghai, China); adenosine and thymidine were purchased from Fluorochem Ltd. (Derbyshire, UK). All the chemicals were guaranteed reagents and were used without further purification. Commercial laboratory filter paper (quantitative ashless) was purchased from Hangzhou Xinhua Paper Industry Co., Ltd. (China). The water used in all the experiments was purified by using a Milli-Q Advantage A10 system (Millipore, Bedford, MA, USA) with a resistivity higher than 18.2 MΩ·cm.

### Fabrication of the paper-based Ag-NP/cellulose-NF composites

The silver nanoparticles (Ag-NPs) were deposited onto the surface of the cellulose nanofibers (NFs) of the filter paper by means of the silver mirror reaction. Briefly, 2.50 mL of fresh aqueous NaOH solution (5.0 wt %) was added into 50.0 mL ice-bath cooled fresh aqueous AgNO_3_ solution (3.0 wt %), whereupon the brown AgOH precipitate was formed; afterwards, concentrated aqueous NH_3_ solution was added dropwise under swift stirring into the mixture until the precipitate dissolved. Then, 15.0 mL of aqueous β-ᴅ-glucose solution (10 wt %) was added into the prepared Tollens’ reagent under continuous stirring. The solution obtained was immediately poured into a Petri dish where 5 pieces of filter paper (size 1 × 1 cm^2^) were placed. In order to control the size and final density of the deposited silver nanoparticles, the filter paper pieces were left in the solution for different periods of time (2, 4, 6, 8, and 10 min). The corresponding piece of filter paper was the removed from the solution, thoroughly washed with water and ethanol, and dried in nitrogen flow. The corresponding silver-nanoparticle/cellulose-nanofiber (Ag-NP/cellulose-NF) composites obtained were denoted as Ag-NP/cellulose-NF–A, B, C, D, and E, respectively.

### Characterizations

Direct surface observations of the Ag-NP/cellulose-NF composite sheets by field-emission scanning electron microscopy (FE-SEM) were carried out in a Hitachi SU-70 electron instrument with an EDAX HORIBA X-Max 80006 equipment working at accelerating voltages of 3.0 kV. To prepare the samples for transmission electron microscopy (TEM) measurements, a small piece of the corresponding sample was cut from the sheet and stirred in 5.0 mL of ethanol overnight to yield a suspension, which was dripped onto a carbon-coated copper grid followed and dried in air. TEM and high-resolution TEM (HR-TEM) images were acquired using a Hitachi HT-7700 instrument working at an acceleration voltage of 100 kV and a JEM 2100F electron microscope operated at an accelerating voltage of 200 kV, respectively. To obtain the size distributions of the silver nanoparticles in the samples, the size of 100 randomly selected particles in a TEM images was measured manually. Powder X-ray diffraction (XRD) patterns were acquired on a Philips X’Pert Pro diffractometer with a Cu Kα (λ = 0.15405 nm) radiation source. Diffuse reflectance UV–vis spectra were recorded by using a Shimadzu UV-2450 spectrophotometer in the diffuse-reflectance mode using an integrating sphere accessory with BaSO_4_ as reference. X-ray photoelectron spectra were acquired by using a VG Escalab Mark 2 spectrophotometer equipped with a Mg Kα X-ray source (*h*ν = 1253.6 eV), the peak positions were internally referenced to the C 1s peak at 285.50 eV.

### SERS measurements

The Ag-NP/cellulose-NF composite sheets were used as SERS substrates, and aqueous solutions of R6G with different concentrations (from 1 × 10^−6^ M to 1 × 10^−18^ M) were used as the test samples. 10 μL R6G solution with the given concentration was dripped onto the Ag-NP/cellulose-NF substrate using a pipette; after the solvent was volatilized, the sample was put on a glass slide to carry out the SERS measurement. The spectra were acquired using a Jobin Yvon LabRam HR UV Raman spectrometer, which was operated at an excitation wavelength of 514 nm with a maximum power of 250 mW. The SERS signals were recorded from four randomly selected points on the paper substrate with an integration time of 5 s for each spectrum. To monitor the molecular recognition between adenosine and thymidine, 10 μL aqueous solution (10 µM) of each nucleoside was dripped separately onto two pieces of the paper substrates; after the evaporation of the solvent, 10 μL aqueous solution of the complementary nucleoside was dripped onto the corresponding paper substrate, and then the SERS spectra were measured after drying under the same experimental conditions as noted above. The finite element method (FEM) modeling of the plasmonic properties of the silver nanoparticles of the Ag-NP/cellulose-NF–C substrate was conducted by employing the RF module of Comsol Multiphysics, and the parameters adopted were based on the silver nanostructures from the FE-SEM observation, together with the excitation at 514 nm. The optical constants of metallic silver were acquired from the literature [69].

## Supporting Information

FE-SEM micrographs of the paper-based SERS substrate Ag-NP/cellulose-NF–E; histograms of the silver nanoparticle size distribution and EDX spectra of the samples Ag-NP/cellulose-NF–A, –B, and –C; SERS spectra of R6G at different concentrations obtained by using substrate Ag-NP/cellulose-NF–E.

File 1 Additional figures.
