# Supplementary material for: A silver-nanoparticle/cellulose-nanofiber composite as a highly effective substrate for surface-enhanced Raman spectroscopy
Source: Beilstein J Nanotechnol. 2019 Jun 24;10:1270–9. doi: 10.3762/bjnano.10.126 (PMC6604729; doi:10.3762/bjnano.10.126)
Supplement: File 1 — Additional figures. [file Beilstein_J_Nanotechnol-10-1270-s001.pdf]

BEILSTEIN JOURNAL OF NANOTECHNOLOGY

Supporting Information

for

A silver-nanoparticle/cellulose-nanofiber composite as a highly effective substrate for surface-enhanced Raman spectroscopy

Yongxin Lu, Yan Luo, Zehao Lin and Jianguo Huang

Beilstein J. Nanotechnol. **2019,** *10,* 1270–1279. doi:10.3762/bjnano.10.126

Additional figures

License and Terms: This is a supporting information file under the terms of the Creative Commons Attribution License (http://creativecommons.org/licenses/by/4.0). Please note that the reuse, redistribution and reproduction in particular requires that the authors and source are credited.
The license is subject to the *Beilstein Journal of Nanotechnology* terms and conditions: (https://www.beilstein-journals.org/bjnano)

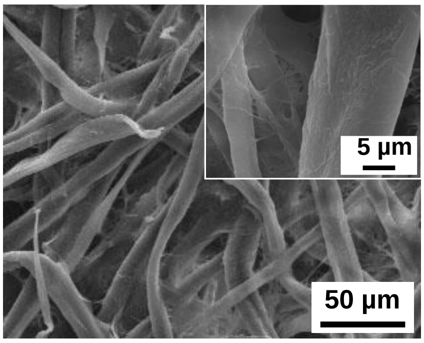

Figure S1: FE-SEM image of bare cellulose laboratory filter paper, showing microfiber assemblies, the inset represents an enlarged image of the microfibers.

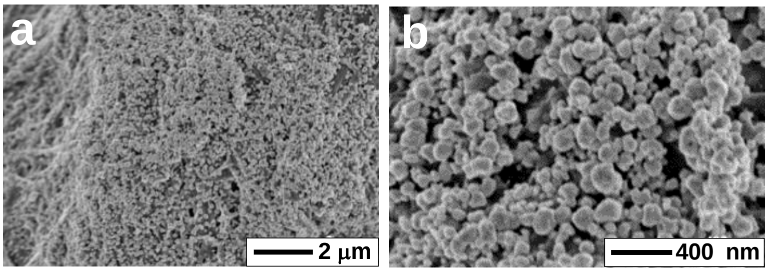

Figure S2: FE-SEM micrographs of the paper based SERS substrate Ag-NP/cellulose-NF–E which was fabricated with a reaction time of 10 minute.

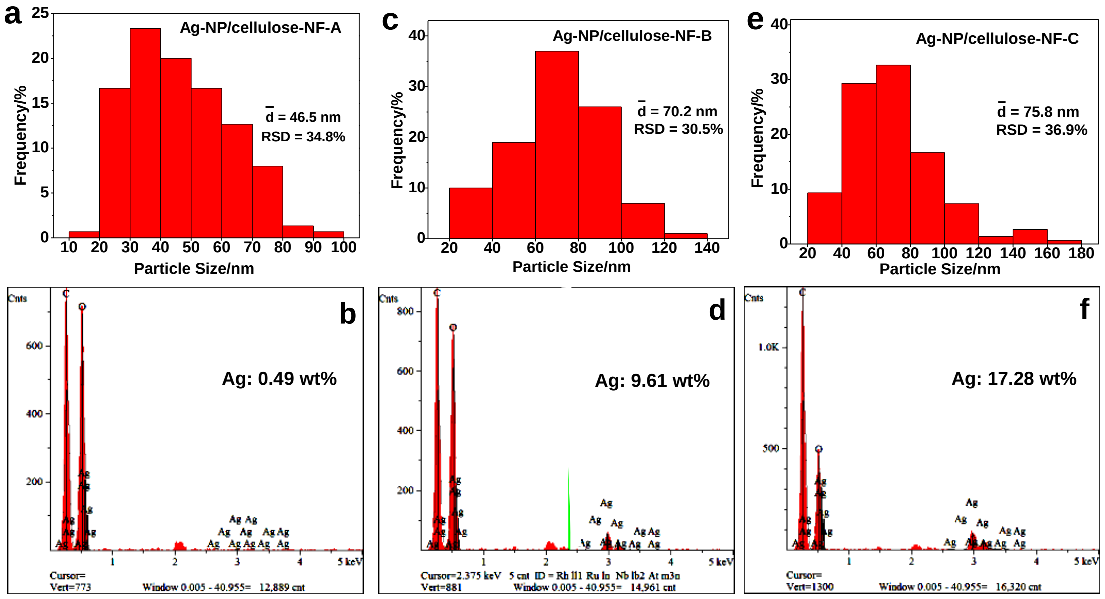

Figure S3: Histograms of the silver nanoparticle size distribution and energy dispersive X-ray (EDX) analysis reports of the paper based SERS substrates Ag-NP/cellulose-NF–A (a,b), –B (c,d), and –C (e,f), which were fabricated with reaction time of 2, 4, and 6 minutes, respectively.

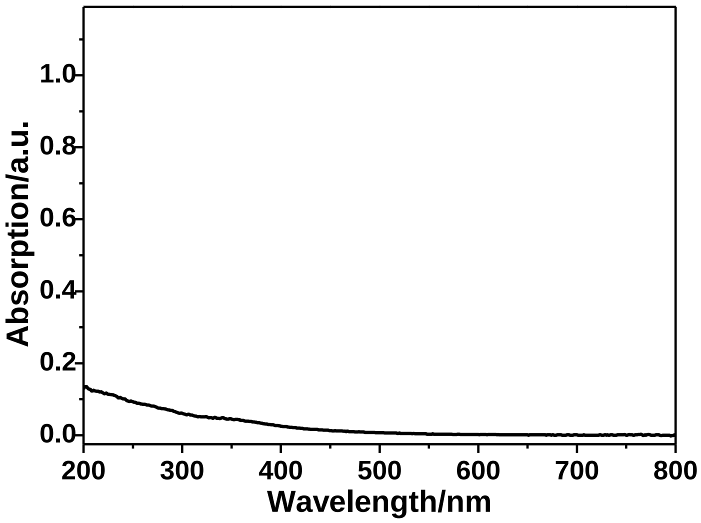

Figure S4: Diffuse reflectance UV–vis spectrum of bare filter paper.

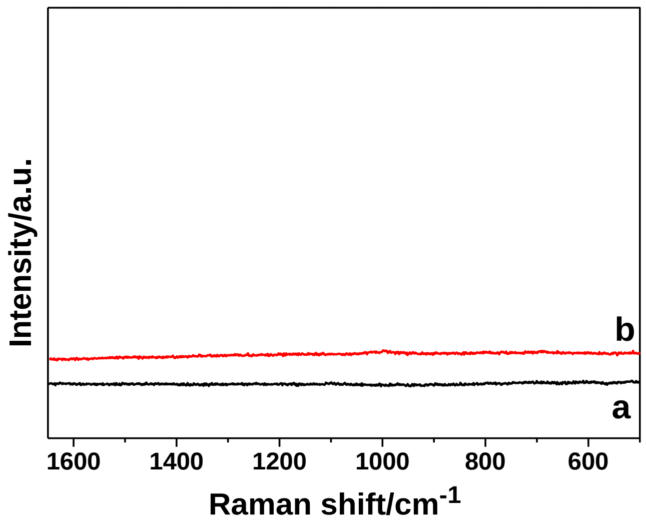

Figure S5: Raman spectra of bare filter paper (a) and the paper based SERS substrate Ag-NP/cellulose-NF–C (b).

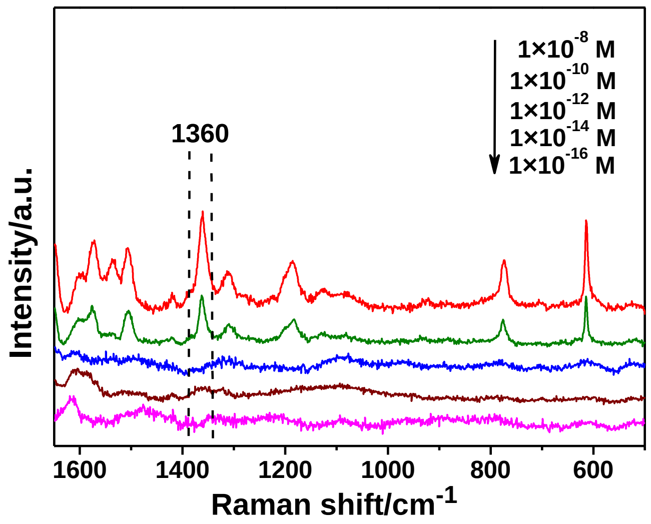

Figure S6: SERS spectra of R6G at various concentrations obtained by employing the paper based substrate Ag-NP/cellulose-NF–E.

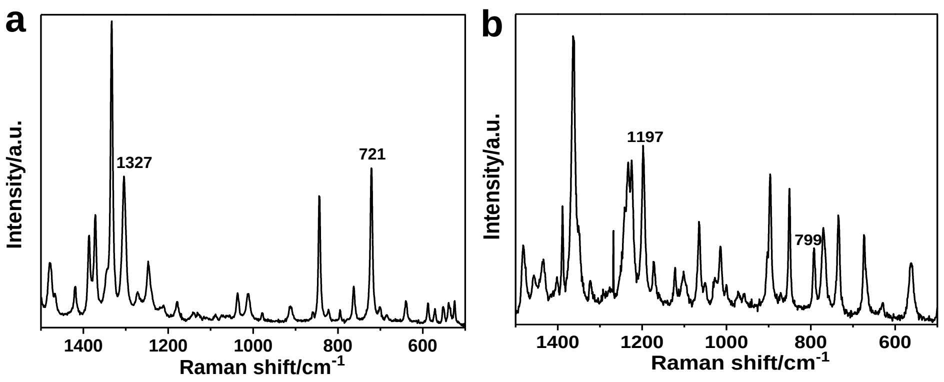

Figure S7: Raman spectra of bulk adenosine (a) and thymidine (b) powders.
